# Clinical Features and Treatment Outcome of Acute Promyelocytic Leukemia Patients Treated at Cairo National Cancer Institute in Egypt

**DOI:** 10.4084/MJHID.2011.060

**Published:** 2011-12-07

**Authors:** Ola Khorshid, Amira Diaa, Mohamed Abd El Moaty, Rafat Abd El Fatah, Ihab El Dessouki, Maha Abd El Hamid, Essam El Noshokaty, Ghada El Saied, Tamer M Fouad, Safaa M Ramadan

**Affiliations:** 1 Medical Oncology Department National Cancer Institute, Cairo University; 2 Clinical Pathology Department National Cancer Institute, Cairo University

## Abstract

The current study reports the clinical features and treatment outcome of 67 patients with acute promyelocytic leukemia (APL) treated at National Cancer Institute (NCI-Cairo), in Egypt from January 2007 to January 2011. The median age at presentation was 29 years. Bleeding was the most common presenting symptom (79%). Most patients had an intermediate risk Sanz score (49%) and 34% had a high risk score. The median follow-up time was 36 months. All evaluable patients were treated for induction with the simultaneous administration of all-*trans* retinoic acid (ATRA) and an anthracycline. The original AIDA treatment protocol was modified due to resource limitations at the NCI-Cairo by replacing of idarubicin with daunorubicin or doxorubicin in most of the cases and the inclusion of cytarabine during the consolidation phase only in pediatric patients. All patients who achieved molecular complete remission after consolidation received two-year maintenance treatment with low dose chemotherapy composed of 6 mercaptopurine, methotrexate and intermittent ATRA courses. Five patients died before treatment initiation due to bleeding, three died during induction chemotherapy due to infectious complications (n=2) and bleeding (n=1) and one patient died during consolidation therapy due to infection. The main therapeutic complications during the induction phase were febrile neutropenia (42%), bleeding (18%) and differentiation syndrome (11%). All patients achieved molecular CR at end of consolidation therapy at a median time of 100 days. The 3-year OS was 89%. Two patients relapsed at 13 and 24 months, respectively. Adapting standard AIDA treatment protocols to limited resources by reducing dose-intensity during consolidation, using ATRA in the consolidation phase and alternative anthracyclin (doxorubicin) may be a valid treatment option for APL in developing countries. In spite of the increased incidence of high and intermediate risk disease in our cohort, we reported an acceptable CR rate, toxicity and OS.

## Introduction

Acute promyelocytic leukemia (APL) is a distinct subtype of acute myeloid leukemia characterized by its morphology, t(15;17) translocation leading to PML-RARa fusion gene. APL represents 5–20 % of AML patients.^1^,^2^ In Egypt, leukemia comprises 10% of all malignancies with AML representing 16.9%.^3^ The National Cancer Institute is the 1^st^ and largest comprehensive cancer center in the Egypt and the Middle East. Annually around 19,500 new cancer patients present to the NCI. Leukemia represents 7% of all malignancies.^4^ With the introduction of all-*trans* retinoic acid (ATRA; tretinoin) in the treatment armamentarium of APL, 90% of patients can achieve complete remission and about 70% of them are potentially cured.^5^–^8^

The NCI standard of care for the treatment of APL patients is based on the simultaneous administration of ATRA and an anthracycline based chemotherapy given both at induction and consolidation (AIDA-like protocol).^5^–^9^ Two years of maintenance treatment are offered for all patients achieving molecular complete remission at the end of consolidation. As for the pediatric patients cytarabine (Ara-C) is added to the consolidation treatment. However, two major health care problems exist in Egypt: these are limited resources and lack of non-governmental reimbursement of expensive drugs such as arsenic trioxide for salvage therapy. Hence at the NCI-Cairo, regimens are adopted and modified according to the highest CR, EFS, and the least toxicity profile together with the highest possible cost effectiveness. Therefore, APL treatment protocol was also modified according to the available resources mainly by the replacement of Idarubicin with Doxorubicin and reducing dose intensity in consolidation as detailed in methods below. Here, we report the clinical features and the treatment outcome of APL patients admitted to our center from January 2007- to January 2011 and treated according to NCI-Cairo APL protocol.

## Patients and methods

### Patient selection

Patients diagnosed with APL genetically confirmed by the presence of t(15;17) and/or of the *PML/RARA* fusion by RT-PCR were enrolled. Age, gender, comorbid conditions at presentation, initial WBC, platelet count, Sanz score and FLT3 internal tandem duplication were analyzed. Induction treatment for adult (>18 yrs) patients consisted of 3 doses of intravenous doxorubicin 60mg/m^2^ or idarubicin 12mg/m^2^ D1–3 combined with All Trans Retinoic acid (ATRA) 45mg/m^2^ in 2 divided doses till morphological complete remission or for a maximum of 45 days. At the end of induction therapy the bone marrow for all patients was assessed for morphologic CR after complete recovery of their blood counts. Patients achieving CR received consolidation therapy consisting of 2 cycles of 3 doses of doxorubicin 60mg/m^2^ or idarubicin 12mg/m^2^ D1–3 in combination with ATRA 45mg/m^2^ for 15 days. Patients who did not achieve morphologic CR received 3 further cycles of the same induction regimen.

At end of consolidation therapy, molecular CR (CRm) was assessed initially by FISH and subsequently confirmed by RT–PCR for *PML-RARA* transcript.^10^ All patients achieving molecular CR were offered a 2-year maintenance treatment with 6 mercaptupurine 60mg/m^2^/d, methotrexate 15mg/m2 IM/weekly and ATRA 45mg/m2 D1–14 every 3 months.

For pediatric patients (<18 yrs), the ATRA dose was reduced to 25mg/m^2^ and was given for a max of 60 days during induction in combination with Idarubicin 12mg/m2 at D3, 5, 7 for standard risk patients and at D1, 3, 5 for high risk patients.

Consolidation therapy consisted of two cycles as follows: Consolidation I consisted of ATRA 25mg/m^2^ for 14 days plus high dose Ara-C (1gm /m^2^ dose/12hrs D1–3) and mitoxantrone (10mg/m^2^ D4,5). Consolidation II consisted of ATRA 25mg/m^2^ for 14 days in combination with Idarubicin 5mg/m^2^ dose D1,3,5. Intrathecal Ara-C was given once at each consolidation cycle. High risk patients or patients not achieving molecular CR received a 3^rd^ cycle of the same regimen given at consolidation I.

Maintenance treatment for pediatric APL patients consisted of ATRA 25mg/m2 D1–14, 6 mercaptopurine 50mg/m2/d and methotrexate 25mg/m^2^ weekly for 2 years. Intrathecal Ara-C was given every 3 months for 1 year to all patients.

Supportive therapy was given to all patients during the entire duration of induction. Platelets and fresh frozen plasma were transfused to maintain platelets count > 50 × 10^9^/l and fibrinogen >150 mg/dl. Packed red cell concentrates were given to maintain Hb level > 8 gr/dl. During induction therapy, prednisone at the dose of 0.5 mg/kg/d was administered to prevent APL differentiation syndrome in patients with WBC ≥10× 10^9^/L. Once APL differentiation syndrome was suspected, ATRA treatment was temporarily discontinued and dexamethasone given i.v. at 10 mg/12 hrs for 4 days or until disappearance of symptoms and signs of the syndrome.

### Outcome definitions and statistical analysis

Hematologic CR (HCR) was defined as the reconstitution of normal marrow cellularity with less than 5% leukemic promyelocytes, together with PB cell counts of polymorphonuclear leukocytes (PMN) > 1,500/μL and platelets >100,000/μL. Molecular remission (CRm) was defined as the disappearance, on an ethidium bromide-stained electrophoresis gel, of the specific *PML/RARA* amplification band identified at diagnosis. Overall survival (OS) was defined as the time from the diagnosis to death from any cause. For the statistical analysis, (SPSS 15.0 software, USA) was used. Numerical data were described in terms of means and medians for central tendency and standard deviation and range, minimum and maximum for dispersion. Overall survival was determined using the Kaplan-Meier product limit method.

## Results

### Baseline clinical and laboratory characteristics

From January 2007 to January 2011, 67 genetically confirmed APL patients were diagnosed at the Medical Oncology Department, National Cancer Institute Cairo University, Egypt.

The baseline characteristics of these patients are summarized in Table 1. The median age was 29 years (range, 3–72), 53 patients were 18 years of age or older and 14 patients were younger than 18 years. There was a slight male predominance (53%). Patients presenting with APL related complications were 80% of the total. These were mainly in the form of bleeding (79%) and febrile neutropenia (5%) while positive hepatitis C virus as a comorbid condition was seen in 1.5% of patients. Blood and platelet transfusion as supportive therapy were given to all patients as indicated. According to Sanz score (5), patients were mainly in the intermediate risk (49%) and 32% were of high risk. FLT3-ITD mutation was assessed in only 14 patients of whom 3 were positive.

### Treatment outcome

Only 62 patients received induction therapy. Of them 51 received the adult APL protocol and 11 received the pediatric APL protocol as described above. The remaining 5 patients were considered as failures due to early death within three days of admission and before induction. All of them died due to intracranial hemorrhage. Complications during induction were documented in 41 patients(66%) and consisted febrile neutropenia 42%, bleeding 18%, differentiation syndrome 11%, and heart failure in 1.6%. Induction mortality was recorded in 3 patients (4.8%) and were due to infection in 2 patients and bleeding in one).

Fifty nine patients received consolidation treatment as planned. One patient died during consolidation therapy due to sepsis. At the end of consolidation, all the 58 evaluable patients achieved complete molecular remission. The median time to complete molecular remission was 100 days. The 3-year OS was 88% and two patients relapsed at 13 and 24 months from CR. They both received the same induction therapy and are alive in CR.

## Discussion

Our results reveal the various challenges faced in countries with limited resources, like Egypt, in the treatment of APL. At the NCI-Cairo adult APL patients are treated with a modified PETHEMA LPA99 regimen with simultaneous administration of doxorubicin and ATRA in the induction and consolidation phases.^9^ Doxorubicin is used as a replacement for Idarubicin, because it is more affordable and readily available. Pediatric APL protocols are also modified to include cytarabine during consolidation therapy. The median age of APL patients in the complete series (adults and pediatrics) was 29 years. Although our population age is slightly younger than what is reported in various prospective trials (about 37–40 years),^8^,^11^ it is similar to the recorded median age (30 years) in another retrospective study by the Tunisian group.^12^ While the predominance of the intermediate risk group is in line with various prospective studies (~50%),^8^,^11^ there was a slightly higher proportion of high risk patients (35%) in the present series compared to most other studies which report a frequency of 25% for this APL subgroup.^8^,^11^ This figure was similar to two retrospective series by the Tunisian and the Brazilian group, who reported 35% and 37%, respectively.^12^,^13^

The overall mortality was 13% and bleeding (67%) was the main cause of death in this series. Early death was the major cause of mortality (12%) and most of these patients (7.5%) died at diagnosis before induction therapy. The reported early death rate in this series is in line with what is reported in two recent studies of population based registries, the Swedish adult acute leukemia registry and the US SEER. Both studies reported a high early mortality rate in APL patients of arround 30% and 17%, respectively.^14^,^15^ In the former study, the main cause of early death was hemorrhage (41%) that occurred within 14 days. Most of the cases also died even before initiation of therapy.^14^

The rationale for avoiding Ara-C in the PETHEMA trial LPA 99 was to reduce death in CR without increasing the incidence of relapse. In fact in the presented series, one patient died during consolidation representing 1.5% of overall mortality similar to the observed rate in the PETHEMA LPA 99 trial (1.3%).^9^ Again, only two patients relapsed and are still alive and in CR after treatment with the same Cairo-NCI induction protocol. The rate of differentiation syndrome (11%) was similar to that in other reports.^8^,^9^,^11^ Despite the limited resources that mandated modifications of the original LPA99 protocol we were able to document a CR rate (100% CRm in evaluable patients) and OS similar to those reported in the PETHEMA LPA99 protocol (>85%).^9^,^11^

We recognize the limitations of our results since this is a retrospective report of a relatively small series of patients observed and treated in a single center. Therefore, these results need to be confirmed prospectively in larger series. However, the CR rate and OS are encouraging and suggest that despite the limited resources available in developing countries APL patients can still achieve outcomes comparable to international standards.

## Figures and Tables

**Figure 1:**
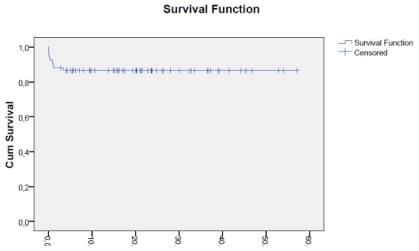
Overall survival of APL patients.

**Table 1:** Patient characteristics.

		N	%
**Total**		67	
**Median age (range)**			

≥18years	33 (18–72)	53	

<18 years:	10.4 (3–16)	14	
**Sex**			

Male		36	53

Female		28	41
**Clinical conditions at Diagnosis**			

Bleeding		53	79

Febrile neutropenia		5	7

HCV		1	1.5

**Transfusion**			

Blood Transfusion		66	98

Platelet transfusion		63	94
**Organomegaly**			

Hepatomegaly		6	9

Splenomegaly		7	10
**WBC count**			

WBC≥10 (x 10^9^/L)		23	34

WBC<10 (x 10^9^/L)		44	66
**Platelet Count**			

≥40 (x 10^9^/L)		16	24

<40 (x 10^9^/L)		51	76
**Sans Risk category**			

Low risk		11	16

Intermediate risk		33	49

High Risk		23	34

**Total**		67	
**Bone Marrow Cellularity**			

Hypocellular		26	39

Hypercellular		17	25

Normal cellular		24	36

**FLT3-ITD (only in 14 patients)**			

Wild		11	79

FLT3-ITD mutation		3	21
**Induction Treatment**	**n=62**		

Doxorubicin or Idarubicin + ATRA		51	82

Pediatric protocol		11	18
**Induction Complications**			

Bleeding		11	18

Febrile neutropenia		26	42

Differentiation syndrome		7	11

Heart failure		1	1.6
Death during induction		3	4.47
**Consolidation regimen**	**n=59**		

Doxorubicin + ATRA		47	80

Pediatric protocol		12	20
**Consolidation Complication**			

Febrile neutropenia		9	15

Death		1	1.49
**Maintenance treatment**	**n=58**		
**Median survival mo**		19.23	
(95% CI)		(16.33–23.75)	

**Table 2:** Causes of death.

	APL (N=67)	
	N	%
**Total deaths**	9	13.43
**Causes of death**		
**- Early before induction**	5	7.46
Bleeding	5	
**- Induction Mortality**	3	4.47
Bleeding	1	
Febrile neutropenia	2	
**- Consolidation mortality**	1	1.49
Febrile neutropenia	1	
